# SARS-CoV-2 specific T cell and humoral immune responses upon vaccination with BNT162b2: a 9 months longitudinal study

**DOI:** 10.1038/s41598-022-19581-y

**Published:** 2022-09-14

**Authors:** Junko S. Takeuchi, Ami Fukunaga, Shohei Yamamoto, Akihito Tanaka, Kouki Matsuda, Moto Kimura, Azusa Kamikawa, Yumiko Kito, Kenji Maeda, Gohzoh Ueda, Tetsuya Mizoue, Mugen Ujiie, Hiroaki Mitsuya, Norio Ohmagari, Wataru Sugiura

**Affiliations:** 1 Department of Academic-Industrial Partnerships Promotion, Center for Clinical Sciences, National Center for Global Health and Medicine, Tokyo, 162-8655 Japan; 2 Department of Epidemiology and Prevention, Center for Clinical Sciences, National Center for Global Health and Medicine, Tokyo, 162-8655 Japan; 3 Department of Laboratory Testing, Center Hospital of the National Center for the Global Health and Medicine, Tokyo, 162-8655 Japan; 4 AIDS Clinical Center, National Center for Global Health and Medicine, Tokyo, 162-8655 Japan; 5 Department of Refractory Viral Infection, Research Institute, National Center for Global Health and Medicine, Tokyo, 162-8655 Japan; 6 Division of Antiviral Therapy, Joint Research Center for Human Retrovirus Infection, Kagoshima University, Kagoshima, 890-8544 Japan; 7 Division of Core Diagnostics, Abbott Japan LLC, Tokyo, 108-6305 Japan; 8 Disease Control and Prevention Center, National Center for Global Health and Medicine, Tokyo, 162-8655 Japan; 9 Center for Clinical Sciences, National Center for Global Health and Medicine, 1-21-1, Toyama, Shinjuku-Ku, Tokyo, 162-8655 Japan

**Keywords:** Vaccines, Viral infection, Viral infection, Epidemiology

## Abstract

The humoral and cellular immune responses against severe acute respiratory syndrome coronavirus 2 (SARS-CoV-2) upon the coronavirus disease 2019 (COVID-19) vaccination remain to be clarified. Hence, we aimed to investigate the long-term chronological changes in SARS-CoV-2 specific IgG antibody, neutralizing antibody, and T cell responses during and after receiving the BNT162b2 vaccine. We performed serological, neutralization, and T cell assays among 100 hospital workers aged 22–73 years who received the vaccine. We conducted seven surveys up to 8 months after the second vaccination dose. SARS-CoV-2 spike protein-specific IgG (IgG-S) titers and T cell responses increased significantly following the first vaccination dose. The highest titers were observed on day 29 and decreased gradually until the end of the follow-up period. There was no correlation between IgG-S and T cell responses. Notably, T cell responses were detected on day 15, earlier than the onset of neutralizing activity. This study demonstrated that both IgG-S and T cell responses were detected before acquiring sufficient levels of SARS-CoV-2 neutralizing antibodies. These immune responses are sustained for approximately 6 to 10 weeks but not for 7 months or later following the second vaccination, indicating the need for the booster dose (i.e., third vaccination).

## Introduction

The coronavirus disease 2019 (COVID-19), caused by severe acute respiratory syndrome coronavirus 2 (SARS-CoV-2), has imposed a tremendous burden on global morbidity and mortality since December 2019^1^. In response to the COVID-19 pandemic, multiple vaccines have been rapidly developed and tested for their efficacy and safety. BNT162b2, an mRNA-based SARS-CoV-2 vaccine (Pfizer-BioNTech), was the first COVID-19 vaccine listed for emergency use and COVAX roll-out by the World Health Organization, with a 95% efficacy in clinical trials^2^. To examine the effectiveness of the vaccine, a growing body of studies has investigated SARS-CoV-2-specific humoral and cellular responses following the vaccination. Studies have shown that a two-dose regimen of BNT162b2 induces SARS-CoV-2 specific humoral and cellular responses, such as immunoglobulin G (IgG) antibodies^3–5^, neutralizing antibodies^6–8^, as well as CD4^+^ and CD8^+^ T cells^6,7,9^.

However, some important issues remain to be addressed. First, little is known about neutralizing antibodies and cellular immune responses against SARS-CoV-2 following the COVID-19 vaccination compared to the evidence on IgG antibodies. Second, epidemiological evidence on temporal changes in humoral and cellular responses and their associated factors, and use of longitudinal data with repeated measures, is limited. Third, studies on the correlation between humoral and cellular responses are scarce. Investigation of humoral and cellular immune responses during and after the COVID-19 vaccination regimen is required to understand its effectiveness, the duration of effectiveness, and the differences in such responses across varying background factors.

Hence, this longitudinal study aimed to investigate chronological changes in IgG antibody, neutralizing antibody, and T cell responses, along with their associated factors, during and after the BNT162b2 vaccination regimen, in hospital workers of a national medical institution in Japan.

## Methods

### Consent statement

Written informed consent was obtained from all the participants before the baseline survey. The study protocol was approved by the Ethics Committee of the National Center for Global Health and Medicine (NCGM), Japan (approval number: NCGM-A-004175-00). All methods were performed in accordance with the relevant guidelines and regulations. The funders did not play any role in the conduct of this study.

### Study design and participants

We performed a longitudinal study among the staff of NCGM, a national medical institution designated for research and treatment of infectious diseases, between March and December 2021, after the start of the vaccination program. We recruited 100 volunteers via e-mail from NCGM staff aged 20 years or above who were scheduled to receive the first COVID-19 vaccination dose on the dates of the baseline survey. None of the participants were on immunosuppressive medication or had a history of COVID-19. All participants received the mRNA-based SARS-CoV-2 vaccine BNT162b2 (Pfizer-BioNTech) according to the standard protocol (two doses of 30 µg administered 3 weeks apart)^6,10^. A total of seven surveys were conducted on the following schedule: day 1 (immediately after the first dose), day 15, day 29 (7 days after the second dose), day 61, day 82–96 (at the time of periodic health checkup), day 224–232, and day 263 (~ 8 months after the second dose).

### Cells and viruses

VeroE6_TMPRSS2_ cells^11^ were obtained from the Japanese Collection of Research Bioresources (JCRB) Cell Bank (Osaka, Japan). VeroE6_TMPRSS2_ cells were maintained in DMEM supplemented with 10% FCS, 100 μg/mL penicillin, 100 μg/mL streptomycin, and 1 mg/mL G418. The SARS-CoV-2 NCGM-05-2N strain (SARS-CoV-2_05-2N_) was isolated from the nasopharyngeal swab of a patient with COVID-19 admitted to the NCGM hospital^8,12^.

### Serological assays

We performed three serological assays to detect the IgM against SARS-CoV-2 spike protein (IgM-S) and IgG antibodies against the receptor-binding domain (RBD) of the SARS-CoV-2 spike protein and nucleocapsid protein (IgG-S-RBD and IgG-N, respectively). The presence of IgG-N antibodies can indicate SARS-CoV-2 infection prior to the study and during follow-up, regardless of the vaccination status. In contrast, the presence of IgM-S and IgG-S-RBD indicates the previous infection and/or humoral immunity following the vaccination, because BNT162b2 is constructed to express the full-length spike protein.

To detect IgG-S-RBD, the AdviseDx SARS-CoV-2 IgG II assay was performed using the Abbott ARCHITECT^®^ following the manufacturer's protocol. The assay detects the IgG antibodies against the receptor-binding domain (RBD) of the S1 subunit of the SARS-CoV-2 spike protein using chemiluminescent microparticle immunoassay (CMIA). The resulting chemiluminescence in relative light units (RLU) indicates the strength of the response, which in turn reflects the quantity of IgG-S-RBD present. We considered 50 AU/mL and above as the cutoff for seropositivity, as recommended by the vendor. We also tested 4160 AU/mL as the cutoff, given that this threshold may correspond to a 95% probability of sufficient neutralizing activity^13^. This quantitative IgG-S-RBD test could evaluate an individual’s humoral response to vaccines.

To detect IgM-S, the AdviseDx SARS-CoV-2 IgM assay, based on semi-quantitative CMIA, was performed using the Abbott ARCHITECT^®^ according to the manufacturer's instructions. This assay is designed to detect IgM to the spike protein of SARS-CoV-2. We defined the seropositive cutoff as 1.0 (index value) and above, according to the vendor’s recommendation.

For the IgG-N assay, the Abbott ARCHITECT^®^ SARS-CoV-2 anti-N IgG assay based on semi-quantitative CMIA was performed. We considered the index values of 1.40 and above as seropositive cutoff according to the manufacturer’s instructions.

### Neutralization assay

An enzyme-linked immunosorbent assay (ELISA)-based semi-quantitative neutralization assay was conducted at three points of the survey (day 15, 29, and 61). It was performed at a single serum dilution according to the manufacturer’s instructions (SARS-CoV-2-NeutraLISA, Euroimmun, Germany). Antibody-mediated inhibition of soluble human receptor protein (angiotensin-converting enzyme 2, hACE2) binding to the RBD of the viral S1 region was tested. The surrogate neutralizing capacity was calculated as the percentage of inhibition (% IH): IH = 100% − (extinction of the respective sample × 100%/extinction of the blank). An IH < 20% was considered as negative, IH ≥ 20% to < 35% intermediate, and IH ≥ 35% positive. Each serum sample was tested in duplicates.

The in vitro virological experiment-based neutralizing activity was determined by quantifying the serum-mediated suppression of the cytopathic effect (CPE) of the SARS-CoV-2 strain in VeroE6_TMPRSS2_ cells, as previously described^8^, but with minor modifications. In brief, each serum was four-fold serially diluted in the culture medium. The diluted sera were incubated with 100 TCID_50_ (50% tissue culture infectious dose) of viruses at 37 °C for 20 minutes. Then, the serum-virus mixtures were inoculated into VeroE6_TMPRSS2_ cells (1.0 × 10^4^/well) in 96-well plates. After culturing the cells for 3 days, the CPE levels observed in SARS-CoV-2-exposed cells were determined by the WST-8 assay using the Cell Counting Kit-8 (Dojindo, Kumamoto, Japan). The serum dilution that resulted in 50% inhibition of CPE was defined as a 50% neutralization titer (NT_50_). Each serum sample was tested in duplicates.

### IFN-γ whole blood assay

SARS-CoV-2 specific CD4^+^ and CD8^+^ T cell responses were investigated at six points (day 1, 15, 29, 61, 224–232, and 263). We quantified T cell-produced interferon-gamma (IFN-γ) in response to SARS-CoV-2 spike peptides using QuantiFERON SARS-CoV-2 RUO (QIAGEN, Germany) according to the manufacturer's instructions. Briefly, 5 mL of whole blood specimens were collected in a collection tube containing sodium heparin (VP-H050K, Terumo, Japan) at six-time points during the vaccination regimen. The collected blood samples were stored at room temperature for no more than 2–3 hours, and 1 ml of the sample was transferred to each QuantiFERON blood collection tube: Nil (negative control), mitogen (positive control), Ag1 and Ag2 (spike protein antigens). Ag1 tube contains CD4^+^ epitopes derived from the S1 subunit (RBD) of the Spike protein, and Ag2 tube contains CD4^+^ and CD8^+^ epitopes from the S1/S2 subunits of the Spike protein^14^. The four tubes were inverted to coat the sides of the tubes and placed in a 37 °C incubator for 16–24 hours. Subsequently, the plasma samples were harvested following centrifugation, stored at − 80 °C, and tested for the release of IFN-γ from stimulated T cells by QuantiFERON ELISA with microplate reader SpectraMax iD5 (Molecular Devices, USA). The quantitative IFN-γ concentration (IU/mL) in each tube was analyzed using the QuantiFERON R&D Analysis Software (version 5.3.0). Nil and mitogen tubes measure background IFN-γ response and nonspecific T-cell response, respectively. In contrast, Ag1 and Ag2 tubes measure RBD-specific CD4^+^ T cell response and S1/S2 subunits specific CD4^+^ and CD8^+^ T cell responses, respectively. The levels of IFN-γ response were obtained by subtracting Nil from Ag1 (Ag1-Nil) or Ag2 (Ag2-Nil). Greater than or equal to 0.20 IU/mL IFN-γ concentration was used to define positive T cell responses, as previously reported^14^.

### Statistical analysis

To examine the difference in changes of IgG-S-RBD titers across background factors (age, sex, and body mass index [BMI]), we fitted the mixed model of repeated measures with an unstructured covariance matrix. Background factors were treated as categorical variables in the models: age (< 40 or ≥ 40 years), sex (men or women), and BMI (< 25 or ≥ 25 kg/m^2^). Log-transformed IgG-S-RBD titers were used as the dependent variable. We set each background factor, time, and interaction terms as fixed factors, and individual identifiers and time as random factors in the model. In all models, we adjusted for age and sex as covariates. We estimated the mean log-transformed IgG-S-RBD titers with 95% confidence intervals (CIs); then, we back-transformed them and presented them as geometric means titers. To compare the mean differences of IgG-S-RBD titers between background factors at each time point, we used the Wald test. As three participants received their second vaccination on an irregular schedule (more than 4 weeks after the first dose), we treated their antibody titers data from day 29 to day 263 as missing values. We repeated these analyses for spike-specific T cell responses.

To examine the correlation between IgG-S-RBD titers and spike-specific T cell responses at each time point (day 1, 15, 29, 61, 224–232, and 263), we conducted Spearman’s rank correlation test. We also run the Spearman’s rank correlation test to examine the correlation of in vitro virological experiment-based neutralizing antibody titers with NeutraLISA and IgG-S-RBD titers.

Statistical analyses were conducted using STATA version 17.0 and GraphPad Prism 9.3.0.

## Results

### Study participants

Table 1 presents participants’ background information. The participants included 32 men and 68 women with a median age of 42 (interquartile range [IQR]: 33–51) years. Major occupations included administrative staff (19%), nurses (17%), doctors (7%), allied healthcare professionals (4%), and other occupations (e.g., researchers and research assistants) (53%). Throughout the seven surveys, the number of participants were 100, 100, 97, 97, 96, 66, and 19 (14 for the tests for T cell responses) for day 1, 15, 29, 61, 82–96, 224–232, and 263, respectively (Table 2).

**Table 1:** Participants’ characteristics (n = 100).

	Overall (n = 100)	
	Median (IQR)	%
**Age, years**	42 (33–51)	
< 40		42
≥ 40		58
**Sex**		
Men		32
Women		68
**Job category**		
Doctors		7
Nurses		17
Allied healthcare professionals		4
Administrative staff		19
Others (e.g., researchers and research assistants)		53
**Department**		
COVID-19-related department		6
The other medical department		31
Non-medical department		63
**Body mass index, kg/m**^**2**^	21.8 (19.9–23.3)	
< 25		83
≥ 25		17

IQR: interquartile range.

**Table 2:** Humoral and cellular immune responses during and after BNT162b2 mRNA-based SARS-CoV-2 vaccination regimen.

	Days after first vaccine dose						
	Day 1 (n = 100)	Day 15 (n = 100)	Day 29 (n = 97)	Day 61 (n = 97)	Day 82–96 (n = 96)	Day 224–232 (n = 66)	Day 263 (n = 19^§^, 14^||^)
**IgG-S-RBD (AU/mL)**	2.6	550*	20582*^†^	9649*^‡^	5419*^‡^	780*^‡^	784*^‡^
	(1.43–4.40)	(277–1013)	(11372–29747)	(5168–13143)	(2854–8209)	(439–1345)	(301–1038)
Seropositive (≥ 50)	0%	99%	100%	100%	100%	100%	100%
Seropositive (≥ 4160)	0%	0%	96%	81%	58%	0%	0%
**IgG-N (S/C)**	0.07	0.07	0.08	0.05	0.07	0.07	0.02
	(0.02–0.18)	(0.02–0.17)	(0.03–0.19)	(0.02–0.11)	(0.02–0.14)	(0.03–0.14)	(0.02–0.04)
Seropositive (≥ 1.40)	0%	0%	0%	0%	0%	0%	0%
**IgM-S (S/C)**	0.04	0.79*	2.78*^†^	0.75*^‡^	–	0.11*^‡^	0.15*^‡^
	(0.03–0.08)	(0.45–1.44)	(1.51–4.51)	(0.48–1.52)	–	(0.07–0.29)	(0.08–0.25)
Seropositive (≥ 1.00)	0%	43%	91%	40%	–	3%	0%
**IFN-γ CD4**^+^ **Ag1-Nil (IU/mL)**	0.00	0.85*	5.76*^†^	1.58*^‡^	–	0.42*^‡^	0.25*^‡^
	(− 0.01 to 0.01)	(0.37–1.80)	(2.98–11.29)	(0.57–3.22)	–	(0.15–1.32)	(0.08–0.88)
Positive (≥ 0.20)	0%	84%	100%	91%	–	67%	57%
**IFN-γ CD4**^+^ **and CD8**^+^ **Ag2-Nil (IU/mL)**	0.00	1.72*	9.68*^†^	2.76*^‡^	–	0.91*^‡^	0.48*^‡^
	(− 0.01 to 0.01)	(0.58–3.17)	(5.38–16.85)	(0.85–6.57)	–	(0.28–1.96)	(0.24–1.78)
Positive (≥ 0.20)	0%	92%	100%	96%	–	88%	86%
**NeutraLISA (inhibition %)**	–	29*	99*^†^	98*^‡^	–	–	–
	–	(13–45)	(98–100)	(93–99)	–	–	–
Positive (≥ 35)	–	37%	100%	100%	–	–	–

Data are shown as median (interquartile range).

*P < 0.01 (Day 1 vs Day 15, 29, 61, 82–96, 224–232, or 263).

^†^*P* < 0.01 (Day 15 vs Day 29).

^‡^*P* < 0.01 (Day 29 vs Day 61, 82–96, 224–232, or 263).

^§^The number of participants tested for humoral responses (IgG-S-RBD, IgG-N, and IgM-S).

^||^The number of participants tested for T cell responses (Ag1-Nil and Ag2-Nil).

### Antibody titers and neutralizing activity

**Figure 1:**
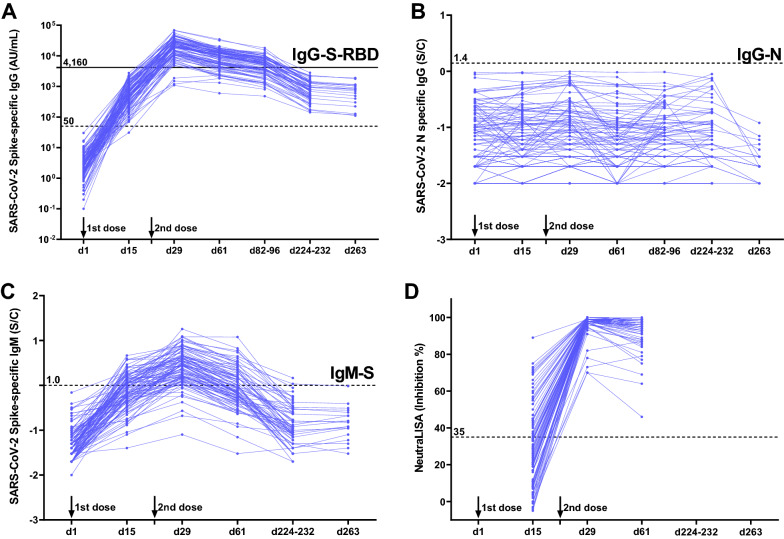
Humoral responses during and after BNT162b2 mRNA-based SARS-CoV-2 vaccination regimen. (**A**–**C**) Humoral responses on day 1 (immediately after the first dose) (n = 100), day 15 (n = 100), day 29 (7 days after the second dose) (n = 97), day 61 (n = 97), day 82–96 (n = 96), day 224–232 (n = 66), and day 263 (n = 19). IgG antibodies against SARS-CoV-2 spike protein (**A**), nucleocapsid (**B**), and IgM against spike protein (**C**) were measured using chemiluminescent microparticle immunoassay (CMIA). An ELISA-based semi-quantitative neutralization assay was also performed (**D**). Dashed lines indicate the cutoff for each assay. All data were represented as line graphs using GraphPad Prism 9.3.0. Black line indicates 4160 AU/mL as the cutoff, given that this threshold may correspond to a 95% probability of sufficient neutralizing activity.

Table 2 and Fig. 1A–C show the humoral response during and after the vaccination regimen. All participants were seronegative for IgG-S-RBD, IgG-N, and IgM-S on day 1. IgG-S-RBD titers were significantly higher than the baseline value in the later time points. We observed the highest IgG-S-RBD titers on day 29 (7 days after the second dose), with a median of 20582 AU/mL (IQR: 11372–29747), followed by a time-dependent decrease: median of 9649 (IQR: 5168–13143) on day 61, 5419 (IQR: 2854–8209) on day 82–96, 780 (IQR: 439–1345) on day 224–232, and 784 (IQR: 301–1038) on day 263. For IgG-S-RBD, 99% (99/100) of the participants were seropositive on day 15 following the first dose, and all participants were seropositive on day 29 (7 days after the second dose) and remained positive during follow-up until day 263. Regarding the surrogate measures of neutralizing activity defined by the IgG-S-RBD titers (cutoff of 4160 AU/mL, a threshold previously shown to correspond to a 95% probability of sufficient neutralizing activity^13^), no participants were seropositive on day 15, while 96% became positive on day 29; this decreased to 81% and 58% on day 61 and day 82–96, respectively. With longer follow-up, all eligible participants became seronegative on day 224–232 and day 263 (< 4160 AU/mL). We also tested surrogate neutralizing activity using the ELISA-based semi-quantitative neutralization assay, which showed that 37% of the participants had sufficient neutralizing activity on day 15, while all participants had sufficient levels of neutralizing activity on day 29 and 61 (Table 2 and Fig. 1D).

To evaluate the results of the ELISA-based semi-quantitative neutralization assay (SARS-CoV-2-NeutraLISA kit, Euroimmun), an in vitro virological experiment-based 50% neutralization titers (NT_50_) was performed using 36 serum samples (12 samples × 3 time points). Although all samples were evaluated as positive for surrogate neutralizing activity by NeutraLISA on days 29 and 61, the serum dilution that resulted in NT_50_ spanned a wide range of values (174 to 4585-fold and 145 to 2312-fold dilution for days 29 and 61, respectively) as shown in Supplementary Fig. S1A. We also tested the correlations between NeutraLISA and NT_50_ (Supplementary Fig. S1A) and between IgG-S-RBD titer and NT_50_ (Supplementary Fig. S1B). On day 15, neither NeutraLISA nor IgG-S-RBD titers were correlated with NT_50_. On days 29 and 61, NeutraLISA and IgG-S-RBD titers were significantly correlated with NT_50_, although the results of NeutraLISA appeared to be saturated at around 1000 titers of NT_50_.

**Figure 2:**
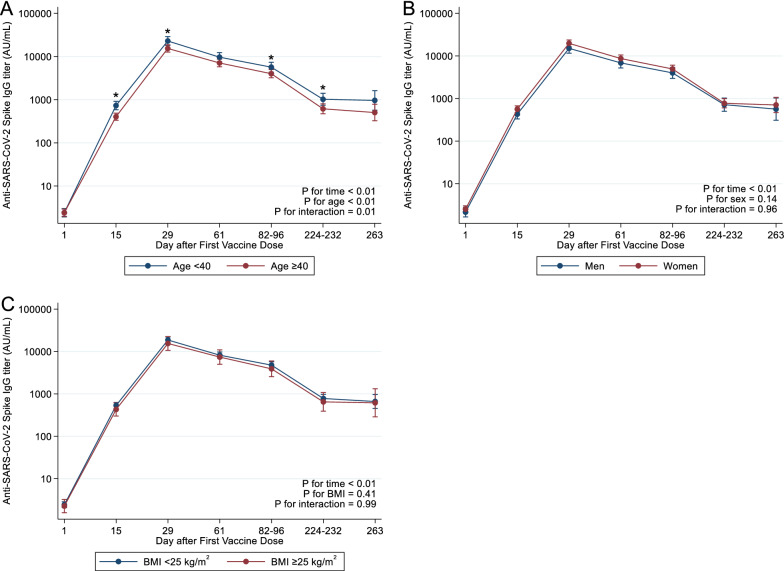
SARS-CoV-2 spike specific IgG titers by background factors. Wald test was used to compare the mean differences of IgG-S-RBD titers between background factors at each time point using STATA version 17.0. (**A**) SARS-CoV-2 spike specific IgG titers by age (< 40 or ≥ 40 years), (**B**) SARS-CoV-2 spike specific IgG titers by sex (men or women), (**C**) SARS-CoV-2 spike specific IgG titers by BMI (< 25 or ≥ 25 kg/m^2^). SARS-CoV-2 spike specific IgG titers were measured on the following schedule: day 1 (immediately after the first dose) (n = 100), day 15 (n = 100), day 29 (7 days after the second dose) (n = 97), day 61 (n = 97), day 82–96 (n = 96), day 224–232 (n = 66), and day 263 (n = 19).

There was a significant association of younger age (< 40 years) with higher IgG-S-RBD titers throughout the five survey time points (*P* < 0.01) (Fig. 2A and Supplementary Table S1). Although we did not observe a statistically significant difference in IgG-S-RBD titers with respect to sex or BMI, females or individuals with normal BMI tended to have higher IgG-S-RBD titers than males or those with BMI ≥ 25 kg/m^2^ throughout the surveys (Fig. 2B,C and Supplementary Table S1).

### Vaccine-induced T cell responses

**Figure 3:**
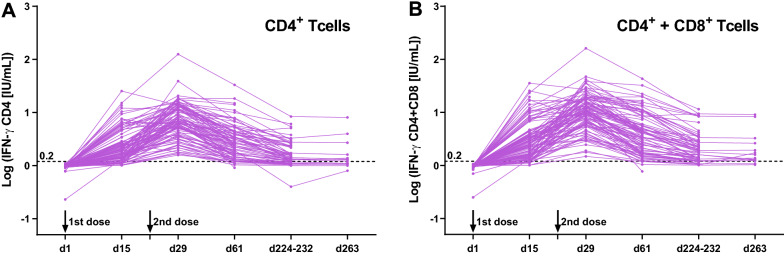
SARS-CoV-2 spike specific T cell response during and after BNT162b2 mRNA-based SARS-CoV-2 vaccination regimen. T cell responses on day 1 (immediately after the first dose) (n = 100), day 15 (n = 100), day 29 (7 days after the second dose) (n = 97), day 61 (n = 97), day 224–232 (n = 66), and day 263 (n = 14). The release of IFN-γ from stimulated CD4^+^ T cells (**A**) and both CD4^+^ and CD8^+^ T cells (**B**) with SARS-CoV-2 spike peptides using QuantiFERON SARS-CoV-2 RUO. All data were represented as line graphs using GraphPad Prism 9.3.0. Dashed lines indicate the cutoff for the assay.

Table 2 and Fig. 3 show the cellular response during and after the vaccination regimen. None of the participants had T cell responses against SARS-CoV-2 antigens on day 1. Following the first vaccination dose, T cell responses increased (0.85 and 1.72 IU/mL for Ag1-Nil and Ag2-Nil, respectively) and peaked after the boost on day 29 (5.76 and 9.68 IU/mL for Ag1-Nil and Ag2-Nil, respectively), but decreased to day 61 (1.58 and 2.76 IU/mL for Ag1-Nil and Ag2-Nil, respectively), day 224–232 (0.42 and 0.91 IU/mL for Ag1-Nil and Ag2-Nil, respectively), and day 263 (0.25 and 0.48 IU/mL for Ag1-Nil and Ag2-Nil, respectively). Overall, higher values for T cell response were obtained for Ag2-Nil (CD4^+^ and CD8^+^) than Ag1-Nil (CD4^+^), indicating that both CD4^+^ and CD8^+^ T cells serve as cellular immunity induced by the COVID-19 vaccination.

While analyzing the association between background factors (age, sex, and BMI) and T cell responses, we did not observe a statistically significant difference in IFN-γ produced by T cells by age or BMI at all time points (data not shown). While men were significantly associated with a higher IFN-γ production by CD4^+^ T cells on day 29 (*P* < 0.05), this association was not observed when one high outlier was excluded (Supplementary Fig. S2).

**Figure 4:**
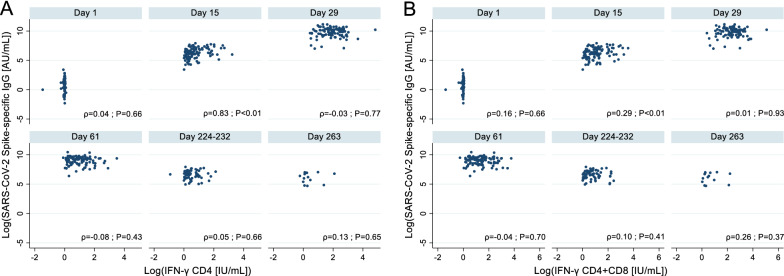
Correlation between SARS-CoV-2 spike specific IgG titers and T cell responses at each time point. Spearman’s rank correlation test was conducted using STATA version 17.0. (**A**) Correlation between IgG-S-RBD titer and spike-specific CD4^+^ T-cell responses. (**B**) Correlation between IgG-S-RBD titer and spike-specific CD4^+^ and CD8^+^ T cell responses. The correlation was assessed at each time point: day 1 (immediately after the first dose) (n = 100), day 15 (n = 100), day 29 (7 days after the second dose) (n = 97), day 61 (n = 97), day 224–232 (n = 66), and day 263 (n = 14).

Further, we found no correlations between IgG-S-RBD and IFN-γ production in CD4^+^ (Fig. 4A) and CD4^+^  + CD8^+^ T cells (Fig. 4B) throughout the surveys, except on day 15, and significantly weak correlations were observed on day 15 (Spearman rank coefficient [rho] = 0.83; p < 0.01 and rho = 0.29; p < 0.01 for CD4^+^ and CD4^+^  + CD8^+^, respectively).

## Discussion

In this study, we documented the IgG-S-RBD antibody and T cell responses on day 15 following the first dose of the BNT162b2 vaccine. All participants were seronegative for both antibody titers and T cell responses against SARS-CoV-2 antigens on day1. These observations were consistent with the fact that none of the participants had a history of COVID-19. We found that both IgG-S-RBD antibody and T cell responses peaked 7 days after the second dose (day 29) and gradually declined until the end of the follow-up period. When 4160 AU/mL was used as the cutoff, sufficient levels of the IgG-S-RBD antibody were maintained for over 6 to 10 weeks following the second dose of BNT162b2 vaccination, but it became seronegative 7 months or later after the second dose among those who were followed up until the last wave of follow-up (n = 19). It is noteworthy that T cell responses were detected earlier than the neutralizing activity. However, we did not observe any statistically significant correlation between humoral and cellular immune responses throughout the survey period.

We observed that IgG-S-RBD titers on day 15 and day 29 were 211-fold and 7916-fold higher, respectively, than that of day 1, and it decreased by 53.1%, 73.7%, and 96.2% on day 61 (39 days after the second dose), day 82–96 (60–74 days after the second dose), and day 263 (8 months after the second dose), respectively, compared to the peak on day 29. Similar to our findings on chronological changes in IgG-S-RBD titers, a multicenter prospective study among health care professionals at multiple time points (day 0, 14, 28, 42, 56, and 90) showed that the antibody response (measured as IgG-S-RBD) was detected on day 14 following the first dose and reached a peak between day 14 and 28 (the second dose was administered on day 21). Further, the response decreased by 43% on day 90 compared to the highest antibody response^3^. As for longer follow-up time period, another study in a large cohort of 4868 participants showed that IgG-S-RBD titers decreased by 94.5% and waned at 6 months after the second dose compared to the peak^15^, which is consistent with our finding.

In contrast to the IgG-S-RBD antibody response, the majority of participants did not attain sufficient levels of neutralizing antibodies against SARS-CoV-2 on day 15: only 37% were positive when assessed by ELISA-based neutralization assay; and 0% were positive when assessed by IgG-S-RBD titers with a cutoff of 4160 AU/mL. However, nearly 100% of participants were positive for neutralizing activity in both assays on day 29. These results suggest that individuals might attain sufficient neutralizing antibodies against SARS-CoV-2 7 days after the second dose of the BNT162b2 vaccine. Further investigations should be tested in neutralizing activity for a longer follow-up time period, given that the proportion of those with sufficient neutralizing antibodies declined after the peak when it was assessed with IgG-S-RBD titers.

In the analysis of investigating the changes of IgG-S-RBD titers across background factors, younger age was significantly associated with higher IgG-S-RBD titers throughout the follow-up period. This finding accords with previous studies that showed the association between age and antibody responses^5,16,17^. It has been suggested that age is an important contributing factor for vaccine-induced humoral immune responses. Similarly, our findings and previous evidence suggest that older individuals are vulnerable to lower IgG-S-RBD titers throughout and after the COVID-19 vaccination compared to younger individuals.

The present study showed that T cell responses were detected on day 15 following the first dose of the vaccination, similar to the antibody responses. This early induction of T cell responses agrees with the findings of a cohort study of 20 Singapore healthcare workers who received the BNT162b2 vaccine^7^, and a study of individuals who received the Moderna mRNA-1273 vaccine^14^. Notably, we observed that T cell responses were detectable earlier than the neutralizing antibodies, consistent with a previous report^7^. A clinical trial evaluating the efficacy of BNT162b2 indicated that the vaccine might prevent the onset of COVID-19 approximately 12 days after the first dose^10^. These observations suggest that early T cell responses, detected on day 15 and earlier than neutralizing antibodies, may contribute to the vaccine’s efficacy. Interestingly, early T cell responses have also been reported in COVID-19 cases. A review paper by Bertoletti et al. suggested that T cells are detected within 7 to 10 days after infection^18^. Moreover, early induction of IFN-γ-producing SARS-CoV-2-specific T cells was observed in patients with mild symptoms^19^. Rapid and functional induction of T cell immune responses may play a critical role in both COVID-19 cases and vaccinated individuals.

However, we did not find any evidence of an association between background factors (age, sex, and BMI) and T cell responses. A recent study reported that the production of IFN-γ from SARS-CoV-2 spike-specific T cells was lower in older vaccinated participants (≥ 80 years old)^20^. In our study, all participants were younger than 80 years old (the maximum age was 73 years), which might explain the null finding on the association between age and T cell responses.

We did not observe any statistically significant correlation between IgG-S-RBD and IFN-γ production in CD4^+^ and CD4^+^  + CD8^+^ T cells. Some participants had higher IFN-γ values with low or moderate IgG-S-RBD levels and vice versa. However, the correlation between SARS-CoV-2 spike-specific humoral and cellular immune responses remains controversial^7,21^. This discrepancy can be explained by the pre-existence of cross-reactive memory T cells against SARS-CoV-2. Interestingly, previous studies have reported that long-lasting memory T cells, induced by common cold coronaviruses, can display robust cross-reactivity to SARS-CoV-2^22,23^. However, the participants in this study did not show T cell responses against SARS-CoV-2 spike antigens on day 1, implying that none of the participants had pre-existing cross-reactive memory T cells against SARS-CoV-2.

The strengths of the present study include the longitudinal design with repeated measures of antibodies and T cell responses during and after the COVID-19 vaccination regimen. However, this study has several limitations. First, our study was conducted in a small-scale cohort of hospital workers in a single medical institution in Japan; thus, our findings might not be generalized to other settings. Second, the sufficient level of neutralizing activity or cellular immunities that is enough to protect against SARS-CoV-2 infection is not well known. In this study, sufficient neutralizing activity levels were defined as IH ≥ 35% in the ELISA-based semi-quantitative neutralization assay (NeutraLISA), and the positive results evaluated by NeutraLISA spanned a wide range of values when the samples were analyzed by an in vitro virological experiment (summarized in Supplementary Fig. S1). While sufficient neutralizing activity was also defined as cutoff of 4160 AU/mL^13^ on IgG-S-RBD titers in this study, we could not validate the threshold using our data. Further studies are warranted to determine sufficient levels of SARS-CoV-2 IgG antibody, neutralizing antibody, and T cell responses against SARS-CoV-2 infection and the duration of immunity induced by the vaccine. Finally, we could not assess the neutralizing activity with NeutraLISA after day 61; thus, it is unknown whether the neutralizing capacity is waning at the later timepoint. Nonetheless, similar studies of health care workers consistently reported that neutralizing activity elicited by two vaccine doses reached peak titer around 2 to 4 weeks and then decreased^8,15^.

In conclusion, our study demonstrated that BNT162b2 vaccination induces SARS-CoV-2 specific IgG-S-RBD antibody and T cell responses, which were detected on day 15 following the first dose and earlier than the onset of neutralizing activity, peaked after the second dose and gradually declined over time. Sufficient levels of these responses against SARS-CoV-2 infection were maintained for over 6 to 10 weeks but not for 7 months or later following the second dose of BNT162b2 vaccination, which may indicate the need for the booster dose. Our study contributes to a better understanding of the humoral and cellular immune responses upon the COVID-19 vaccination.

## Supplementary Information

Supplementary Information.

## Data Availability

The data are not publicly available due to ethical restrictions for public deposition but available from the Center for Clinical Sciences, National Center for Global Health and Medicine, Tokyo, Japan (corresponding author: Wataru Sugiura, wsugiura@hosp.ncgm.go.jp) for researchers who meet the criteria to access the data.
