# Supplementary material for: SARS-CoV-2 specific T cell and humoral immune responses upon vaccination with BNT162b2: a 9 months longitudinal study
Source: Sci Rep. 2022 Sep 14;12:15447. doi: 10.1038/s41598-022-19581-y (PMC9472721; doi:10.1038/s41598-022-19581-y)
Supplement: Supplementary file 1 — Supplementary Information. [file 41598_2022_19581_MOESM1_ESM.docx]

**Supplementary Information for**

**SARS-CoV-2 specific T cell and humoral immune responses upon vaccination with BNT162b2**

**: A 9 months longitudinal study**

Junko S. Takeuchi*^1^, Ami Fukunaga*^2^, Shohei Yamamoto*^2^, Akihito Tanaka^3^, Kouki Matsuda^4^, Moto Kimura^1^, Azusa Kamikawa^1^, Yumiko Kito^1^,

Kenji Maeda^4^, Gohzoh Ueda^5^, Tetsuya Mizoue^2^, Mugen Ujiie^6^, Hiroaki Mitsuya^4^, Norio Ohmagari^6^, Wataru Sugiura^7^

**Affiliations:**

^1^ Department of Academic-Industrial Partnerships Promotion, Center for Clinical Sciences, National Center for Global Health and Medicine, Tokyo 162-8655, Japan

^2^ Department of Epidemiology and Prevention, Center for Clinical Sciences, National Center for Global Health and Medicine, Tokyo 162-8655, Japan

^3^ Department of Laboratory Testing, Center Hospital of the National Center for the Global Health and Medicine, Tokyo 162-8655, Japan

^4^ Department of Refractory Viral Infection, Research Institute, National Center for Global Health and Medicine, Tokyo 162-8655, Japan

^5^ Division of Core Diagnostics, Abbott Japan LLC, Tokyo 108-6305, Japan

^6^ Disease Control and Prevention Center, National Center for Global Health and Medicine, Tokyo 162-8655, Japan

^7^ Center for Clinical Sciences, National Center for Global Health and Medicine, Tokyo 162-8655, Japan

*Equal contribution as first author

**Corresponding Author**

Wataru Sugiura

Center for Clinical Sciences, National Center for Global Health and Medicine, 1-21-1, Toyama, Shinjuku-ku, Tokyo 162-8655, Japan

TEL:  +81-3-3202-7181, Fax: +81-3-5273-6850

E-mail: [wsugiura@hosp.ncgm.go.jp](mailto:wsugiura@hosp.ncgm.go.jp)

**This PDF file includes:**

Supplementary Table S1

Supplementary Figures S1 and S2

**Supplementary Table S1. Estimated geometric means with 95% confidence intervals of SARS-CoV-2 spike specific IgG titers by background factors**

Data are shown as geometric means with 95% confidence intervals estimated by the repeated measures mixed model.

All models were adjusted for age (<40 or ≥40 years) and sex.

*Significant differences between groups at each time point (*P*<0.05).

GMT: geometric mean titers.

**Supplementary Figure S1. Scatter plot of 50% Neutralization titers with NeutraLISA (A) or IgG-S titers (B)**

An *in vitro* virological experiment-based neutralizing assay was performed using 36 serum samples (12 samples × 3 time points). Spearman’s rank correlation test was conducted using STATA version 17.0. (A) Correlation between the results of the ELISA-based semi-quantitative neutralization assay (SARS-CoV-2-NeutraLISA kit,Euroimmun) and an *in vitro* virological experiment-based 50% neutralization titers (NT_50_). (B) Correlation between SARS-CoV-2 Spike-specific IgG (IgG-S-RBD) and an *in vitro* virological experiment-based 50% neutralization titers (NT_50_). Red lines indicate the cutoff (35 %) for the NeutraLISA assay.

**Supplementary Figure S2. Estimated geometric means with 95% confidence intervals of IFN-γ CD4^+^ T cell by background factor (sex)**

We fitted a mixed model of repeated measures using an unstructured covariance matrix. Background factors were treated as categorical variables in the models: sex (men or women). Log_10_-transformed IFN*-γ* outcomes were used as dependent variables. We considered each background factor (sex), time, and interaction as fixed factors, and individual identifiers and time as random factors in the model. We estimated the mean log_10_-transformed IgG-S titer with 95% confidence intervals (CIs); then, we back-transformed them and presented them as geometric means. To compare the mean differences of IFN*-γ* outcomes between background factors at each time point, we used the Wald test with Bonferroni adjustment. Blue and red lines indicate men and women, respectively.
